# Partitioning Fraction of Variance Explained into Strong Localized Effects and Weak Diffuse Effects

**DOI:** 10.64898/2026.01.06.697735

**Published:** 2026-01-20

**Authors:** Fang Nan, David Azriel, Armin Schwartzman

## Abstract

High-dimensional genetic data present substantial challenges for estimating the fraction of variance explained (FVE) by genome-wide single-nucleotide polymorphisms (SNPs). Standard approaches for SNP heritability estimation, such as GWAS heritability (GWASH) and linkage disequilibrium score (LDSC) regression, typically assume Gaussian distributions for SNP effect sizes. However, empirical evidence indicates that SNP effects are often heavy-tailed, with a small subset of variants exerting disproportionately large influence. Such settings violate the recently established bounded-kurtosis effect (BKE) condition, under which these FVE estimators are consistent. Consequently, widely used methods may yield severely biased estimates when strong effects are present. We introduce a decomposed FVE estimation framework that accommodates heavy-tailed and heterogeneous SNP effect distributions. The proposed approach partitions total heritability into contributions from strong and weak genetic effects, estimating the former using low-dimensional adjusted R-squared and the latter using an extension of FVE estimation methodology that remains valid under BKE compliance. We further develop a test for detecting violations of the BKE condition and compare several high-dimensional screening procedures for identifying strong-effect SNPs when they are not known in advance. Simulation studies show that the proposed decomposition substantially improves estimation accuracy over existing approaches in the presence of heavy-tailed effects. Application to the Adolescent Brain Cognitive Development (ABCD) Study demonstrates the practical utility of the method, yielding more reliable heritability estimates for the PolyVoxel Score, a neuroimaging-based biomarker linked to iron accumulation. Our results highlight the importance of accommodating effect heterogeneity in large-scale genomic studies.

## Full Text Availability

The license terms selected by the author(s) for this preprint version do not permit archiving in PMC. The full text is available from the preprint server.

